# Rare variants in previously identified linkage regions associated with carotid plaque in Dominican Republic families

**DOI:** 10.1371/journal.pone.0250799

**Published:** 2022-01-12

**Authors:** Nicole D. Dueker, Ashley Beecham, Liyong Wang, Chuanhui Dong, Ralph L. Sacco, Susan H. Blanton, Tatjana Rundek

**Affiliations:** 1 John P. Hussman Institute for Human Genomics, University of Miami, Miami, FL, United States of America; 2 Dr. John T. Macdonald Foundation Department of Human Genetics, University of Miami, Miami, FL, United States of America; 3 Department of Neurology, Miller School of Medicine, University of Miami, Miami, FL, United States of America; 4 Department of Public Health Sciences, Miller School of Medicine, University of Miami, Miami, FL, United States of America; 5 Evelyn F. McKnight Brain Institute, Department of Neurology, University of Miami, Miami, FL, United States of America; University of Colorado Denver - Anschutz Medical Campus, UNITED STATES

## Abstract

Carotid plaque is a subclinical measure of atherosclerosis. We have previously shown measures of carotid plaque to be heritable in a sample of 100 Dominican families and found evidence for linkage and association of common variants (CVs) on 7q36, 11p15, 14q32 and 15q23 with plaque presence. Our current study aimed to refine these regions further and identify rare variants (RVs) influencing plaque presence. Therefore, we performed targeted sequencing of the one LOD unit down region on 7q36, 11p15, 14q32 and 15q23 in 12 Dominican families with evidence for linkage to plaque presence. Gene-based RV analyses were performed using the Sequence Association Test for familial data (F-SKAT) under two filtering algorithms; 1. all exonic RVs and 2. non-synonymous RVs. Replication analyses were performed using a sample of 22 Dominican families and 556 unrelated Dominicans with Exome Array data. To identify additional non-synonymous RVs influencing plaque, we looked for co-segregation of RVs with plaque in each of the sequenced families. Our most strongly associated gene with evidence for replication was *AMPD3* which showed suggestive association with plaque presence in the sequenced families (exonic RV p = 0.003, nonsynonymous RV p = 0.005) and replication families (exonic RV p = 0.04, nonsynonymous RV p = 0.02). Examination of the sequenced family pedigrees revealed two missense variants on chromosome 11 which co-segregated with plaque presence in one of our families; rs61751342 (located in *DENND2B*), and rs61760882 (located in *RNF141*). The rs61751342 missense variant is an eQTL for *SCUBE2* in the atrial appendage. Notably, *SCUBE2* encodes a protein which interacts with vascular endothelial growth factor (VEGF) receptor 2 to regulate VEGF-induced angiogenesis, thus providing biologic plausibility for this gene in atherosclerosis. In conclusion, using targeted sequencing of previously-identified linkage regions, we have identified suggestive evidence for the role of RVs in carotid plaque pathogenesis.

## Introduction

Carotid plaque, a well-established subclinical marker of atherosclerosis, is a known predictor of stroke and myocardial infarction [1]. Heritability studies show evidence for a genetic contribution to plaque [2–5], with heritability estimates ranging from 13%-52% in various populations. A recent study also showed evidence for shared genes between various subclinical measures of atherosclerosis, including plaque, and clinical outcomes [6]. Further evidence for a genetic contribution to plaque comes from genome-wide linkage and association studies. The largest genome-wide association study (GWAS) identified two loci significantly associated with plaque presence (*PIK3CG* and *EDNRA*) and two loci that were suggestively associated *(LDLR*, and *LRIG1)* [7]. Smaller studies of plaque phenotypes have implicated additional regions including 9p21, 10q24 [8] and chromosome 17 [5].

In addition to these studies, we have taken a multi-stage approach to identify genetic variants influencing plaque presence in a large sample of extended families from the Dominican Republic. Within this sample, we have shown plaque presence to be heritable (h^2^ = 50%) and genome-wide linkage analysis identified four chromosomal regions linked to plaque presence; 7q36, 11p15, 14q32 and 15q23. Further, we have shown evidence for the association of common variants (CVs) within these regions [2, 9]. Our current study aimed to further characterize these regions by sequencing the regions under the linkage peaks to identify rare variants contributing to carotid plaque in these families.

## Materials and methods

### Study samples

Study participants were drawn from two studies; the Family Study of Stroke Risk and Carotid Atherosclerosis and the Northern Manhattan Stroke Study (NOMAS), a population-based cohort study. Details of both study designs have been published previously [10, 11]. Briefly, NOMAS enrolled 3,298 stroke-free individuals between 1993 and 2001. A subset of Caribbean Hispanic probands from NOMAS who were at high risk for cardiovascular were subsequently enrolled in the family study. To be eligible for inclusion in the family study, probands needed to have a high risk of cardiovascular disease (defined as 1. Having a sibling with a history of myocardial infarction or stroke, or 2. Having two of the following three risk phenotypes; maximal carotid plaque thickness, left ventricular mass or homocysteine level above the 75^th^ percentile in NOMAS). They also needed to have the ability to provide a family history, obtain family members’ permission to be contacted by research staff, and have at least three first-degree relatives able to participate [10]. Probands for the family study were enrolled in northern Manhattan and family members were enrolled at two locations: New York at Columbia University and in the Dominican Republic (DR) at the Clinicas Corazones Unidos in Santo Domingo. All participants provided written informed consent and the study was approved by the Institutional Review Boards of Columbia University, the University of Miami, the National Bioethics Committee and the Independent Ethics Committee of Instituto Oncologico Regional del Cibao in the DR (20070478 and 20072012).

A total of 34 families with a family-specific LOD score >0.1 at one of the four QTLs for plaque located at chromosomes 7q36, 11p15, 14q32 and 15q23, were selected for our current study; 12 families were sequenced as part of our discovery analyses and 22 families were genotyped on the Exome Array for our replication analyses. Details of the family selection have been published previously [12]. Additional replication analyses were performed using an independent sample of 556 unrelated Dominicans from NOMAS.

### Carotid plaque phenotypes and risk factor measurements

High-resolution B-mode 2-dimensional carotid ultrasound imaging was performed to measure atherosclerotic plaque at the internal and common carotid arteries and bifurcations, according to standardized scanning and reading protocols [13]. Details of our carotid plaque phenotyping have been previously published [2, 13]. Briefly, in-depth imaging of plaques was performed in long axes and multiple angles. The optimized and normalized images were analyzed offline by automated computerized edge detection system M’Ath (Intelligence in Medical Technologies, Inc, Paris, France) and area of each plaque was measured. For each participant, the sum of all plaque areas (mm^2^) within each subject was calculated and expressed as a total carotid plaque area. Plaque presence was then defined as an area of focal wall thickening over 50% greater than surrounding wall thickness in millimeters.

Traditional risk factor data on body mass index (BMI), waist-hip ratio (WHR), diabetes, hypertension and pack-years smoking, was collected during a standardized interview [14]. BMI was defined as weight (kg) divided by height (m)^2^ and WHR was defined as waist circumference divided by hip circumference. Presence of diabetes was defined as fasting blood glucose level ≥126 mg/dl or self-reported history of diabetes. Presence of hypertension was defined as self-reported history of high blood pressure, systolic blood pressure ≥ 140 mmHg, diastolic blood pressure ≥ 90 mmHg, or self-reported use of antihypertensive medication. Pack-years of smoking were calculated as the number of cigarettes smoked per day divided by 20 and then multiplied by the number of years smoking. All data collection procedures were standardized across NOMAS and the Family Study.

### Discovery sample sequencing and quality control

Genomic DNA was isolated from whole blood in family study participants. Targeted -sequencing of the exons in genes within the one LOD unit down region on 7q36 (chr7:150-160Mb), 11p15 (chr11:7–25 Mb), 14q32 (chr14:78–102 Mb), and 15q23 (chr15: 70–95 Mb), as well as targeted sequencing beyond the exons for candidate genes identified previously [9], was performed using a customized Agilent SureSelect Enrichment Kit. Details of our sequencing methods have been previously described [15]. Briefly, DNA libraries were sequenced on an Illumina HiSeq2000 and the Burrows-Wheeler Aligner was used to align the raw sequencing reads to the human reference sequence hg19 [16]. The Genome Analysis ToolKit was used to call variants and potential functional annotations of variants were obtained using ANNOVAR v. 2016Feb01 and SeattleSequation 138.

Quality control was conducted at both variant and sample levels, as described previously [12, 15]. Within each sample, variants having depth <4 or Phred-Like score <100 were set to missing. Variants with VQSLOD <−4 or call rate <75% were removed from analysis. Individuals missing plaque measures (n = 4) were removed prior to segregation analyses and an additional 13 individuals missing covariate values were removed prior to association analyses. All individuals had high concordance (≥95%) between the sequencing data and available genotype data. For the remaining family study samples, pedigree structure was confirmed using the Graphical Relationship Representation software v. 1.2.1.41. Mendelian error checking was performed, and Mendelian errors were set to missing for all the variants called using PLATO v. 0.84 [17].

### Replication sample genotyping and quality control

A total of 698 NOMAS participants and 378 individuals within our 22 replication families were genotyped using the Illumina HumanExome-24v1_B Beadchip, at the Hussman Institute for Human Genomics in the Center for Genome Technology (Miami, FL). Our Exome Array included custom exonic variants selected on the basis of sequencing data obtained in the discovery family data set. Details of the variant selection have been described previously [15].

We removed 39 NOMAS individuals and 18 replication family members due to unexpected duplication or relatedness, gender discrepancy, or low call rate (<98%). Relatedness was assessed in NOMAS using PLINK and all pairs of individuals had pi-hat<0.18. For our current study, individuals missing plaque measures and/or covariate values were also removed prior to association analyses (103 NOMAS participants; 18 replication family members), leaving us with a final sample of 556 Dominican NOMAS participants and 342 replication family members. At the variant level, we removed SNVs with a call rate <95%, monomorphic SNVs and non-exonic SNVs, leaving us with 3,418 exonic rare single nucleotide variants (RVs) in our regions for Exome Array analysis in our replication samples. Mendelian error checking was performed in the replication families and Mendelian errors were set to missing for all the variants called using PLATO v. 0.84 [17].

### Statistical methods

#### Gene-based discovery analyses

Rare SNVs were defined based on frequencies from our NOMAS Dominican participants as previously described [15], with SNVs having an MAF<5% being considered rare. Gene-based analyses were performed using F-SKAT, an extension of the sequence kernel association test for familial samples with binary traits [18]. Two analyses were performed based on ANNOVAR and SeattleSeq annotations; one analyzing all exonic rare variants (UTR 3’, UTR 5’, synonymous, missense, nonsense and splice site variants) and a second restricting analyses to nonsynonymous variants (missense, nonsense and splice site variants). All analyses were adjusted for BMI, waist-hip ratio, diabetes, hypertension, pack-years of smoking and age. Covariates were selected for being previously associated with plaque in a polygenic screen implemented in SOLAR [2]. Our analyses were additionally adjusted for the first principal component obtained via principal components analyses implemented in PC-AiR to account for population substructure [19]. PC-AiR analyses were performed using Exome Array data and were restricted to autosomal variants with call rate > 95% and MAF ≥ 5%. Additionally, variants in pairwise linkage disequilibrium were removed (PLINK option indep-pairwise 100 2 0.8). Gene-based analyses were restricted to RVs with a call rate = 100% as required by F-SKAT and to genes with at least two polymorphic RVs. A p<9.3x10^-5^ was considered significant based on a Bonferroni correction of 534 tests (399 exonic RV genes and 135 nonsynonymous RV genes).

#### Replication analyses

Gene-based RV association analyses using Exome Array data were performed using SKAT-O v. 1.1.2 in the NOMAS sample and F-SKAT v. 1.0 in the replication family sample. These analyses were performed using the same two filtering algorithms employed in the discovery analyses; all exonic RVs and then a subset of nonsynonymous RVs only. Analyses were restricted to genes with at least two polymorphic variants. Family sample replication analyses were further restricted to RVs with call rate = 100% due to requirements of our analysis program, F-SKAT. Replication was defined as genes with *p* < 0.05. All association analyses were adjusted for the same covariates included in our discovery analyses. To account for population substructure in NOMAS, these analyses were adjusted for the first principal component obtained via principal components analyses implemented in Eigenstrat Details of our Eigenstrat analyses and results have been previously reported [20].

#### Segregation analyses

To investigate rare nonsynonymous variants further, we examined discovery family pedigrees with a family-specific LOD score > 0.4 for co-segregation of variants with plaque. Within each family, we filtered to identify variants that were present in all individuals with plaque and were not present in all individuals without plaque.

## Results

### Participant and SNV characteristics

Twelve families containing a total of 205 individuals were included in our discovery analyses. Characteristics of these families are in Table 1.

**Table 1:** Characteristics of the 12 sequenced Dominican families.

		Family-Specific LOD Score											
Family	Individuals per Family	Chr7	Chr11	Chr14	Chr15	% Plaque presence	% Female	% Diabetes	% Hypertension	Age μ ± SD	BMI μ ± SD	Pack-years μ ± SD	WHR μ ± SD
253	13	**0.38**	0.05	-0.05	**0.21**	46.2	61.5	30.8	69.2	49.54 ± 20.72	27.50 ± 4.91	0.88 ± 3.03	0.87 ± 0.07
1917	10	-0.16	**0.54**	**0.14**	0.06	50.0	80.0	20.0	60.0	45.80 ± 16.82	33.79 ± 7.17	12.41 ± 16.88	0.92 ± 0.07
2235	15	0.06	0.04	**0.45**	0.08	33.3	60.0	13.3	60.0	46.80 ± 16.90	28.62 ± 3.85	6.04 ± 11.04	0.92 ± 0.12
2783	13	**0.22**	**0.46**	-0.03	-0.21	23.1	61.6	15.4	61.5	43.31 ± 14.63	28.92 ± 4.91	0.92 ± 1.99	0.93 ± 0.09
3603	12	-0.03	-0.06	0.07	**0.37**	25.0	66.7	8.3	25.0	45.08 ± 20.15	26.56 ± 5.60	4.87 ± 11.08	0.89 ± 0.11
3630	13	0.01	**0.21**	**0.15**	**0.29**	15.4	69.2	0.0	30.8	60.54 ± 15.15	24.68 ± 4.92	5.08 ± 10.83	0.89 ± 0.08
3631	17	**0.14**	-0.03	**0.13**	-0.10	29.4	70.6	11.8	35.3	40.53 ± 15.86	31.53 ± 6.98	4.70 ± 11.07	0.91 ± 0.06
4641	12	0.05	-0.006	**0.28**	0.09	25.0	83.3	58.3	25.0	44.58 ± 17.71	31.75 ± 8.70	5.73 ± 7.00	0.96 ± 0.08
5103	36	0.06	**0.36**	-0.10	-0.25	19.4	36.1	8.3	44.4	43.11 ± 18.41	26.72 ± 5.39	3.43 ± 8.19	0.92 ± 0.07
5279	19	**0.30**	0.06	-0.12	-0.08	52.6	42.1	15.8	42.1	54.42 ± 17.76	25.18 ± 4.17	4.71 ± 14.06	0.95 ± 0.05
5569	17	**0.88**	0.04	**0.27**	0.06	52.9	70.6	5.9	52.9	46.24 ± 18.05	26.64 ± 2.91	7.95 ± 10.21	0.88 ± 0.05
6081	28	**0.19**	**0.78**	-0.30	**1.01**	21.4	60.7	7.1	42.9	47.82 ± 18.53	27.72 ± 5.41	0.38 ± 1.41	0.89 ± 0.09

*Bold family-specific LOD score indicates family was included in discovery analyses for that QTL.

Sequencing within these families identified 3,232 exonic RVs across the four QTLs, as shown in Table 2. Within the chromosome 7 QTL, our smallest sequenced region, 143 polymorphic exonic RVs were identified, of which 11 were novel (7.7%) and 16 were nonsynonymous (11.3%). In the chromosome 11 QTL, 987 exonic variants were identified with 129 being novel (13.0%) and 209 being nonsynonymous (21.2%). Within the chromosome 14 QTL, 856 variants were identified with 109 being novel (12.7%) and 159 being nonsynonymous (18.6%). In the chromosome 15 QTL, our largest sequenced region, 1,246 exonic variants were identified with 150 being novel (12.0%) and 268 being nonsynonymous (21.5%).

**Table 2:** Polymorphic exonic rare variants identified through sequencing, stratified by function*.

	QTL			
Function*	Chr 7	Chr 11	Chr 14	Chr 15
Missense	16	209	157	263
Nonsense	0	0	1	2
Splice site	0	0	1	3
Synonymous	18	168	145	252
UTR 3’ or UTR 5’	90	507	439	619
ncRNA	19	103	113	107
*Total*	*143*	987	*856*	*1*,*246*

*Function based on Annovar and SeattleSeq annotation.

### RV association results

Gene-based analyses were performed under two filtering algorithms; all exonic RVs and then restricted to nonsynonymous RVs. Exonic RV analyses revealed *NRXN3* to be significantly associated with plaque (p = 6.8 x10^-5^), although this finding did not replicate in the replication families (p = 0.82) or NOMAS (p = 0.56). In addition to *NRXN3*, 34 genes showed suggestive association with plaque in the discovery families (S1 Table), of which eight genes showed evidence for replication in the replication families (*AMPD3*, *NOM1*, *MICAL2*, *KCNC1*, *DPP6*, *C11orf58*, *ADM*, *LMO1*) (Table 3). No genes showed evidence for replication in NOMAS (Table 3 and S1 Table). When analyses were restricted to nonsynonymous RVs, five genes showed suggestive association with plaque in the discovery families (p<0.05) and had evidence for replication in replication families (*AMPD3*, *NOM1*, *MICAL2*, *NRDE2* and *FAM181A*) (Table 3). No genes showed evidence for replication in NOMAS (Table 3 and S1 Table).

**Table 3:** Gene-based association results for rare variant analysis in the four linkage regions, for genes with *p <* 0.05 in the discovery families and *p <* 0.05 in the replication families or NOMAS.

			Exonic RVs						Nonsynonymous RVs					
			Discovery Families		Replication Families		NOMAS		Discovery Families		Replication Families		NOMAS	
Gene	Chr	MB Start	# SNVs	Pval	# SNVs	Pval	# SNVs	Pval	# SNVs	Pval	# SNVs	Pval	# SNVs	Pval
*AMPD3*	11	10.47	11	**0.003**	10	**0.04**	16	0.30	4	**0.005**	3	**0.02**	8	0.46
*NOM1*	7	156.74	23	**0.007**	7	**0.003**	30	0.55	6	**0.03**	3	**0.002**	16	0.85
*MICAL2*	11	12.11	15	**0.009**	8	**0.01**	18	0.74	7	**0.007**	6	**0.008**	15	0.83
*KCNC1*	11	17.75	18	**0.01**	8	**4.0x10** ^**-4**^	17	0.35	0	N/A	0	N/A	1	N/A
*DPP6*	7	153.74	14	**0.02**	10	**0.05**	12	0.74	1	N/A	3	0.10	3	0.50
*C11orf58*	11	16.76	8	**0.02**	4	**8.3x10** ^**-4**^	4	0.55	0	N/A	0	N/A	0	N/A
*ADM*	11	10.32	7	**0.03**	4	**0.006**	4	0.28	2	0.21	2	**0.009**	2	0.44
*LMO1*	11	8.24	4	**0.05**	2	**0.02**	2	0.21	0	N/A	1	N/A	1	N/A
*NRDE2*	14	90.74	10	0.07	9	**0.004**	18	0.26	7	**0.03**	6	**6.5x10** ^**-4**^	14	0.08
*FAM181A*	14	94.39	5	0.27	6	**0.01**	5	0.10	2	**0.04**	4	**0.03**	4	0.25

### Segregation analysis results

**Fig 1:**
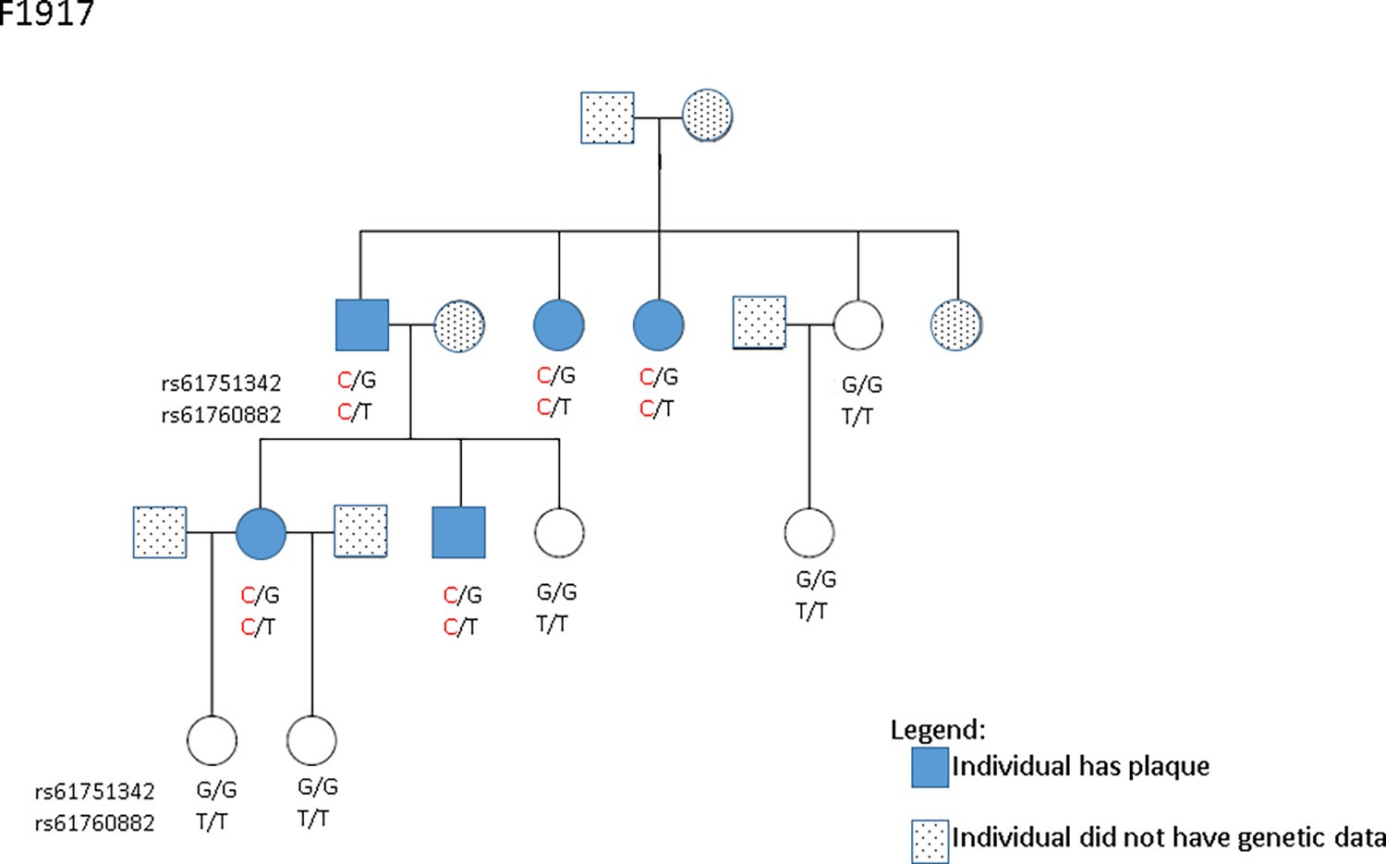
Genotypes for rs61751342 (*DENND2B*) and rs61760882 (*RNF141*), two missense variants found to co-segregate with plaque presence in family 1917.

When examining rare nonsynonymous variants for co-segregation with plaque, we identified two missense variants within the chr11 QTL in F1917: rs61751342 and rs61760882 (Fig 1). The rs61751342 missense variant is located in DENN domain containing 2B gene, *DENND2B*, and is an eQTL for *SCUBE2* in the atrial appendage (*p =* 3.5x10^-5^, GTEx Consortium) and *AKIP1* in subcutaneous adipose tissue (*p =* 2.0x10^-5^, GTEx Consortium). The frequency of the minor allele of rs61751342 in our study was 3.2% in NOMAS and 6.4% in our sequenced families. The following frequencies were observed in other populations according to gnomAD [21]: non-Finnish European = 5.8%, African = 0.95%, Latino = 2.0% and South Asian = 2.5%. The rs61760882 missense variant is located in the ring finger protein 141 gene, *RNF141* and had a minor allele frequency of 0.2% in NOMAS and 6.4% in our sequenced families. The following frequencies of rs61760882 were observed in other populations according to gnomAD: non-Finnish European = 1.8%, African = 0.04%, Latino = 0.07% and South Asian = 0.1%.

## Discussion

Using targeted re-sequencing in extended families from the Dominican Republic, we have identified a suggestive role for RVs within previously-identified linkage regions on chromosomes 7q36, 11p15, 14q32 and 15q23 in carotid plaque. Our study found *AMPD3* to be our most strongly associated gene with evidence for replication in both exonic and nonsynonymous RV analyses, followed by *MICAL2* which was our second most strongly associated gene with evidence for replication in nonsynonymous RV analyses. Notably, these genes showed association with carotid plaque in the replication families but not NOMAS which could reflect several things. First, the associations may be specific to the environmental and genetic context of the families. Families were selected to be at high risk for CVD whereas NOMAS participants were not selected for study inclusion based on CVD risk. Therefore, these RV associations may only be observed in this setting. Second, the association may have been specific to the RVs included in the discovery family analyses. However, in exploratory analyses restricting gene-based analyses to only those variants found in the discovery families, the NOMAS gene associations remained non-significant (S2 Table). Third, the association observed in the discovery families may be a false positive, although association was observed in the replication families lending support to the involvement of *AMPD3* and *MICAL2* in carotid plaque.

*AMPD3* encodes the erythrocyte isoforms of AMP-deaminase. Two additional isoforms of AMP-deaminase are encoded by *AMPD1* and *AMPD2*, of which, the C34T variant in *AMPD1* has been implicated in CVD [22, 23]. Although the role of *AMPD3* in carotid plaque is unknown, previous studies have found common variants in *AMPD3* to be associated with HDL cholesterol [24, 25]. HDL cholesterol is inversely related to CVD risk [26] and may help prevent atherosclerosis [27, 28]. The *MICAL2* gene encodes [F-actin]-monooxygenase MICAL2, a regulator of the SRF signaling pathway. Published GWAS have shown common variants in *MICAL2* to be significantly associated with resting heart rate [29] and white blood cell count [30], traits which can influence atherosclerosis [31] and vascular events [32]. Further, a study investigating gene expression patterns found *MICAL2* to be downregulated in atherosclerotic CAD patients compared to controls [33]. Together, these findings in conjunction with our current study, offer biologic plausibility for *AMPD3* and *MICAL2* in atherosclerosis.

Exonic RV analyses also identified *DPP6* as a candidate gene for carotid plaque presence, a gene we previously identified in common variant analyses [9]. Therefore, the current study validates and extends this finding to suggest that RVs in *DPP6* also play an important role in carotid plaque pathogenesis. The *DPP6* gene encodes dipeptidyl aminopeptidase-like protein 6 which is a ß subunit that conducts the transient-outward K^+^ current I_(to)_ in the human heart [34]. Variants within *DPP6* have been implicated in familial idiopathic ventricular fibrillation [35] and Bazett’s corrected QT intervals (msec) in African Americans [36], a measure which may be a marker for subclinical atherosclerosis [37]. Using these data we can hypothesize that *DPP6* is involved in severe heart disease that is likely caused by gene-related atherosclerotic mechanisms.

Further candidates were identified when examining rare nonsynonymous variants for co-segregation with plaque in individual families. Through this analysis, two missense variants on chromosome 11 were found to co-segregate with plaque. Interestingly, one of these variants, rs61751342 (located in *DENND2B)*, was found to be an eQTL for *SCUBE2* in the atrial appendage. The protein encoded by *SCUBE2* is known to be expressed in vascular endothelial cells and interacts with vascular endothelial growth factor (VEGF) receptor 2 to regulate VEGF-induced angiogenesis [38]. In addition, thrombi commonly form in the atrial appendage in individuals with atrial fibrillation, mitral valve disease and other conditions [39], making this protein a potential therapeutic target for heart disease.

Our study has several limitations. First, only a subset of our extended families were sequenced; however, the selected families showed the highest evidence for linkage to each of our four QTLs. Second, our replication analyses were performed using Exome Array data, therefore, these analyses were restricted to those variants found on the Array. However, we added custom content to our Exome Array which included RVs found in our sequenced DR families but not on the commercially designed array. Fourth, our study population was from the Dominican Republic and therefore findings may not extend to other populations, particularly if the associated RVs have different allele frequencies in other populations [40]. Finally, due to our sample sizes, we had reduced power to detect RV association in the population-based NOMAS cohort, which might attribute to the lack of replication of the family findings in the NOMAS. Study strengths included our carefully defined phenotyping that was performed using standardized protocols, the use of two validation samples and our family study design which minimizes population stratification and increases our ability to investigate RVs.

In conclusion, using a unique sample of extended families from the Dominican Republic, we have extended our previous findings to implicate RVs in previously identified linkage regions in carotid plaque. Our study suggests that rare variation in genes including *AMPD3*, *MICAL2*, *DPP6*, and *DENND2B* may play a role in subclinical atherosclerosis, thereby making them promising novel candidates for the development of anti-atherosclerotic therapies. However, functional studies are warranted to elucidate the specific effects of identified variants on carotid plaque pathogenesis.

## Supporting information

S1 Table Association results for genes with p<0.05 in exonic or nonsynonymous RV analyses in the discovery families. (XLSX)

S2 Table Association results for genes with p<0.05 in exonic or nonsynonymous RV analyses in the discovery families. Replication analyses restricted to only RVs included in discovery family analyses.
(XLSX)
